# Phthiriasis palpebrarum in a 7-year-old girl: a case report

**DOI:** 10.3389/fmed.2025.1716450

**Published:** 2026-01-09

**Authors:** Tingting Cui, Lina Meng

**Affiliations:** Department of Ophthalmology, Qilu Hospital (Qingdao), Cheeloo College of Medicine, Shandong University, Qingdao, China

**Keywords:** blepharitis, case report, pediatric ophthalmology, Phthiriasis palpebrarum, *Pthirus pubis*

## Abstract

This case report presents a rare instance of phthiriasis palpebrarum in a 7-year-old female patient. The patient complained of bilateral eyelid pruritus and erythema lasting for 3 weeks. Slit-lamp examination revealed adult lice and nits adhering to the palpebral margins, and microscopic assessment confirmed the diagnosis of *Pthirus pubis* infestation. Following multiple treatment attempts, all lice and nits were successfully eliminated. With the application of tobramycin eye ointment, the patient achieved full recovery. Phthiriasis palpebrarum is an uncommon condition, especially in pediatric populations, and is frequently misdiagnosed as blepharitis. This case underscores the importance of meticulous clinical examination to prevent misdiagnosis. Complete eradication of lice and nits is critical for the effective management of phthiriasis palpebrarum. For pediatric patients, choosing an appropriate louse removal method is essential to ensure their cooperation during treatment. Furthermore, the patient's family should be provided with detailed guidance on proper hygiene practices to prevent reinfestation.

## Introduction

Phthiriasis palpebrarum is a rare eyelid infestation caused by *Pthirus pubis*. Although *Pthirus pubis* is primarily adapted to colonize pubic hair, it can also infest hair in other anatomical regions, including the scalp, axillae, chest, abdomen, thighs, eyebrows, and eyelashes (1, 2). Notably, eyelash infestation is uncommon even among healthy adults and is particularly rare in pediatric populations. This report describes a case of phthiriasis palpebrarum in a 7-year-old girl, with the aim of enhancing clinical understanding of this rare pediatric manifestation.

## Case description

**Figure 1:**
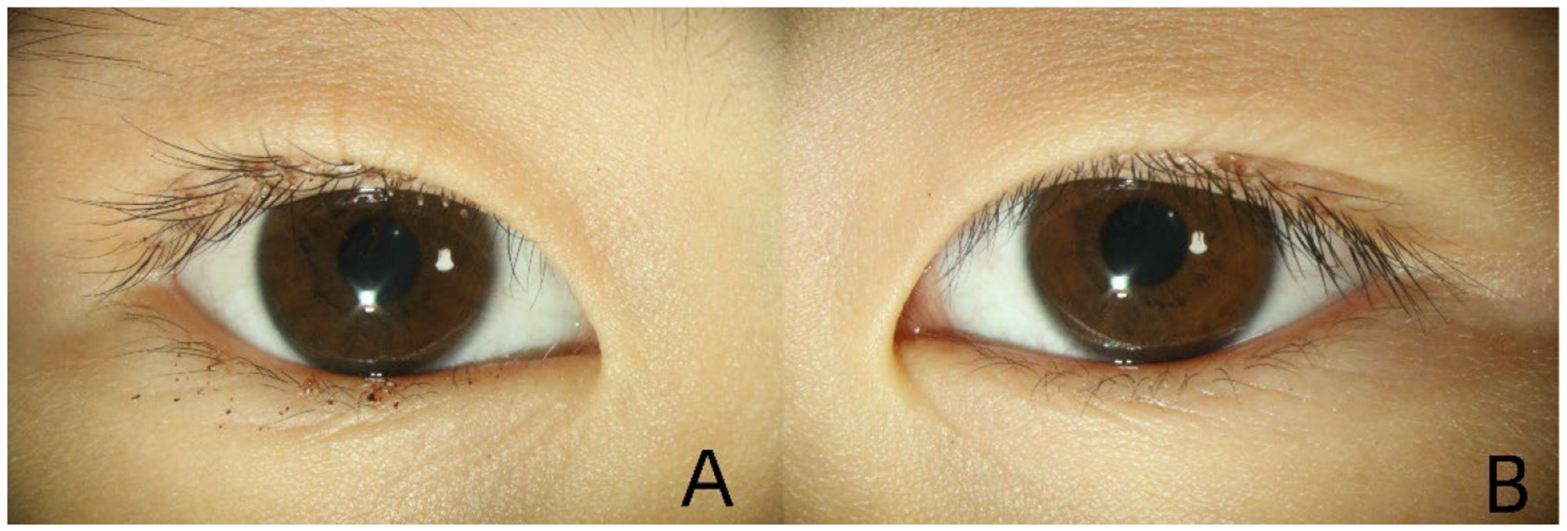
The upper and lower palpebral margins of both eyes exhibited mild erythema and dandruff-like scaling. Scattered petechiae and crusts were observed on the palpebral margins, with greater involvement of the right eye [Figure **(A)** shows the right eye, and Figure **(B)** shows the left eye].

A 7-year-old Chinese girl presented to the Ophthalmology Department of our hospital with bilateral eyelid pruritus and erythema lasting 3 weeks, having received no prior treatment. She had no history of systemic diseases and reported no associated visual impairment. Her distance visual acuity was 20/20 in both eyes. On clinical examination, the upper and lower palpebral margins of both eyes exhibited mild erythema and dandruff-like scaling. Scattered petechiae and crusts were observed on the surface of the palpebral margins (Figure 1).

**Figure 2:**
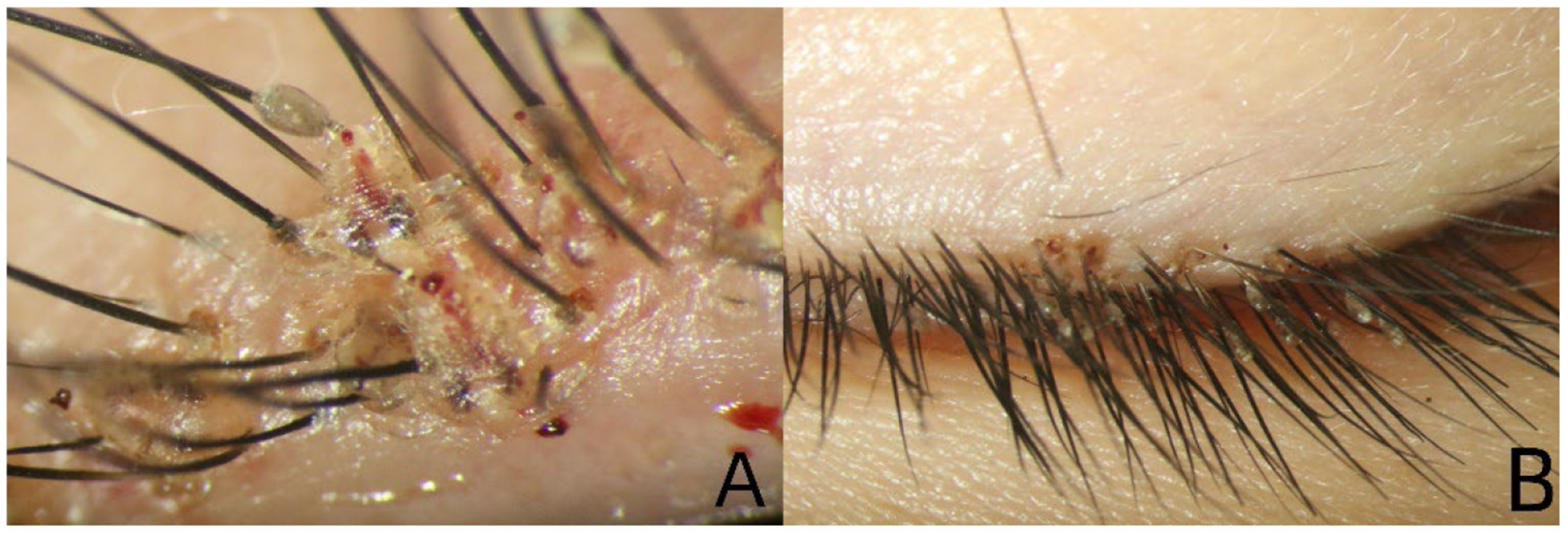
Nits and more than 10 adult lice were adherent to the palpebral margin of the right eye **(A)**. Only nits were observed adhering to the eyelashes of the left eye **(B)**.

Slit-lamp biomicroscopy of the right eye revealed many translucent oval eggs (nits) and more than 10 adult translucent crab-like lice with small spines on their surface, adherent to the palpebral margin (Figure 2A). Some lice were embedded in the palpebral margin skin, while others held onto the root of the eyelashes. One actively moving louse was observed among them. This louse had three pairs of functional legs on each side of its body, with visible blood internally and on its surface. Nits were also attached to the eyelashes. For the left eye, multiple nits were adherent to the eyelashes, although no adult lice were detected (Figure 2B). Neither eye showed conjunctival hyperemia, and the cornea and aqueous humor remained clear bilaterally.

**Figure 3:**
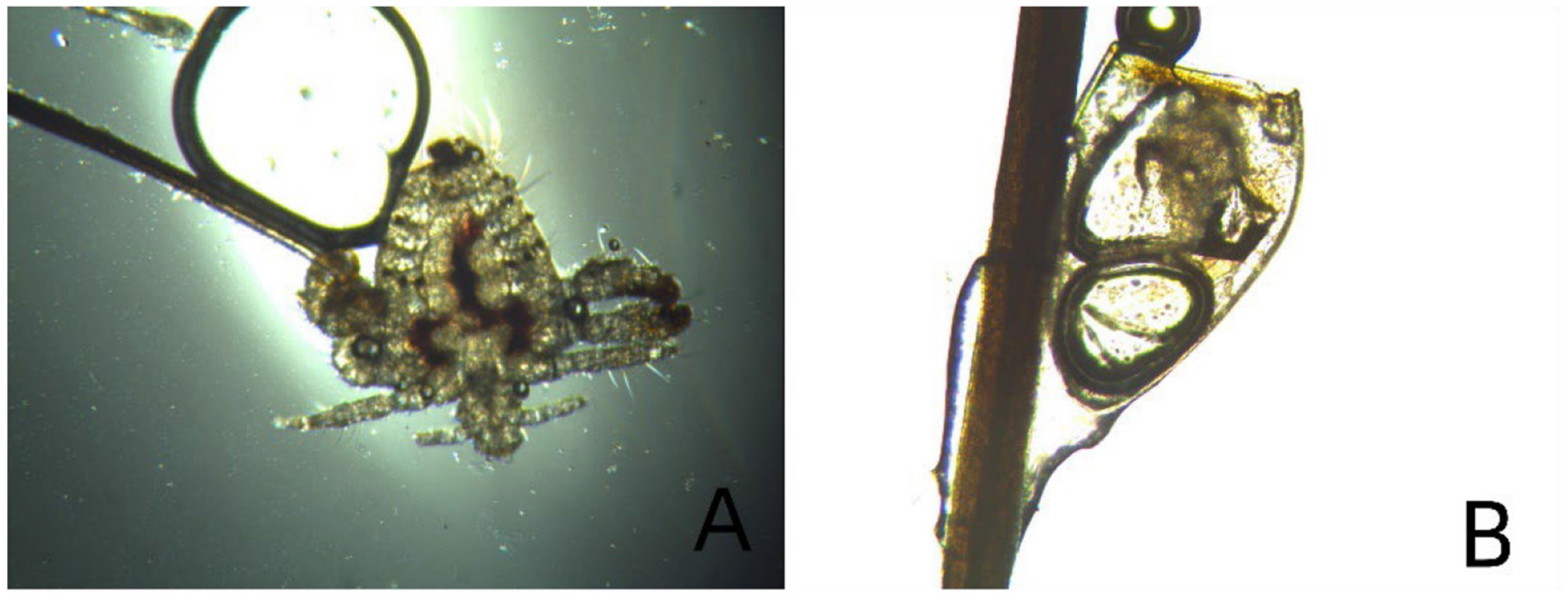
Microscopic findings. **(A)** Depicts an adult *Pthirus pubis* (louse), and **(B)** shows an egg of *Pthirus pubis* (nit).

For confirmatory microscopic examination, a louse clinging to the base of two eyelashes was selected (Figure 3A), as it was more accessible than lice embedded in the skin. The louse was extracted using fine eyelash forceps, and a nit was also subjected to microscopic evaluation (Figure 3B). Microscopic examination confirmed the diagnosis of phthiriasis palpebrarum. No lice or nits were detected in the eyebrows or scalp hair. The girl's parents were questioned regarding potential exposure: the child had no contact with suspicious pets (e.g., stray cats or dogs), and neither the parents nor individuals with whom the child regularly interacted had similar symptoms or a diagnosis of pediculosis pubis. The parents further denied any history of sexual abuse. Despite the administration of topical anesthesia, the child complained of significant pain, accompanied by crying and fussiness during the lice removal procedure. Considerable time and effort were devoted to comforting her and facilitating her cooperation, and all lice and nits were successfully eradicated after multiple attempts. After the successful eradication of all lice and nits, tobramycin eye ointment was prescribed bilaterally three times daily, and the patient's family was counseled on proper hygiene practices. At the 3-week follow-up visit, no lice or nits were detected in either eye, and the patient's symptoms had fully resolved.

## Discussion

Eyelid infestation by *Pthirus pubis* is rare (3). In children, due to the immature development of body hair, *Pthirus pubis* infestation may involve the eyelids and, occasionally, the eyebrows or scalp hair. Pruritus is the primary symptom, often accompanied by a tingling sensation. Adult *Pthirus pubis* closely matches the color of the eyelid skin, making phthiriasis palpebrarum prone to misdiagnosis as seborrheic dermatitis-associated blepharitis, bacterial conjunctivitis, or allergic contact dermatitis (4, 5). In addition to dermoscopy and light microscopy, high-magnification slit-lamp biomicroscopy also aids in differential diagnosis (6). In the present case, the patient's palpebral margins exhibited scattered petechiae and crusts, which were secondary to louse bites and the presence of dark excretory products. This finding may serve as a clinical clue to the presence of blood-sucking parasites.

*Pthirus pubis* infestation can be transmitted via sexual contact, direct contact, and indirect contact. The primary transmission routes include unprotected sexual activity and close personal contact, while indirect transmission may occur through contaminated items such as bed sheets, clothing, and bedpans. It is classified as a sexually transmitted infection (1, 7). The transmission route in this pediatric patient remains unclear. However, child abuse should be considered in pediatric patients who develop this condition. Although non-sexual close-contact transmission of pediatric phthiriasis palpebrarum has been reported, a thorough medical history must be meticulously obtained to identify the infection source. When child sexual abuse is suspected, prompt interdisciplinary collaboration among pediatricians, dermatologists, and social service agencies is essential to protect the child's physical and psychological health.

The key to treating phthiriasis palpebrarum is the complete eradication of all lice and nits. A staged removal approach is recommended: first, lice, nits, and the affected eyelash segments to which they are attached should be excised using microscissors rather than epilated with forceps; subsequently, lice embedded in the palpebral margin skin should be extracted with microsurgical forceps, followed by gentle wiping of the palpebral margin to avoid missing any residual embedded lice. This sequential approach minimizes patient discomfort, thereby improving treatment compliance. For uncooperative pediatric patients, lice removal under general anesthesia may be considered. In addition, for patients in whom physical removal may be incomplete, occlusive ointments such as petrolatum ointment can be applied to the palpebral margin to effectively stifle the lice. In this case, tobramycin eye ointment was topically applied to the patient's eyes. Although this ointment exhibits a weaker occlusive effect on louse respiration compared to occlusive agents such as petrolatum, it still exerts a certain degree of occlusion. Furthermore, it can prevent secondary bacterial infections. Other therapeutic agents for phthiriasis palpebrarum include 0.1%−1% yellow oxide of mercury, 20% sulphacetamide ointment, and 10%−20% fluorescein drops (1). However, for pediatric patients, particular caution should be exercised when selecting medications, with priority given to agents characterized by minimal toxicity and adverse effects.

## Data Availability

The original contributions presented in the study are included in the article/supplementary material, further inquiries can be directed to the corresponding author.
